# Role of Social and Informational Support while Deciding on Pregnancy Termination for Medical Reasons

**DOI:** 10.3390/ijerph15122854

**Published:** 2018-12-14

**Authors:** Kornelia Zaręba, Marta Makara-Studzińska, Michał Ciebiera, Jacek Gierus, Grzegorz Jakiel

**Affiliations:** 1 First Department of Obstetrics and Gynecology, Center of Postgraduate Medical Education, 00-416 Warsaw, Poland; kornelia3@poczta.onet.pl (K.Z.); grzegorz.jakiel1@o2.pl (G.J.); 2 Faculty of Clinical Health Psychology, Jagiellonian University Medical College, 31-501 Krakow, Poland; mmakarastudzinska@gmail.com; 3 Second Department of Obstetrics and Gynecology, Center of Postgraduate Medical Education, 01-809 Warsaw, Poland; 4 Department of Psychiatry, Department of Health Sciences, Medical University of Warsaw, 05-802 Warsaw, Poland; jgierus@gmail.com

**Keywords:** pregnancy termination, abortion, fetal defects, social support, medical community, medical information

## Abstract

*Background*: Poland is a country with restrictive laws concerning abortion, which is only allowed if the mother’s life and health are in danger, in case of rape, and severe defects in the fetus. This paper specifies the forms of support expected by women considering termination from their family, people in their surroundings and professional medical personnel. *Methods*: Between June 2014 and May 2016 patients eligible to terminate a pregnancy for medical reasons were asked to complete an anonymous survey consisting of sixty questions to determine patient profile and forms of support expected from the society, family and professional medical personnel as well as to assess informational support provided. *Results*: Women do not take into consideration society’s opinion on pregnancy termination (95%). The majority of the respondents think that financial support from the state is not sufficient to provide for sick children (81%). Despite claiming to have a medium standard of life (75%), nearly half of the respondents (45%) say that they do not have the financial resources to take care of a sick child. The women have informed their partner (97%) and closest family members (82%) and a low percentage have informed friends (32%). Nearly one third (31%) have not talked to the attending gynecologist about their decision. *Conclusions*: The decision to terminate a pregnancy is made by mature women with a stable life situation—supported by their partner and close family. They do not expect systemic support, as they believe it is marginal, and only seek emotional support from their closest family. They appreciate support provided by professional medical personnel if it is personal.

## 1. Introduction

After being informed about a negative prognosis for a fetus, the parents initially experience mental shock. The next stage is sadness, followed by obtaining information about the nature of the disease affecting the fetus. The psychological burden is very high, as the patient needs to make an independent decision and even submit a written request to have the procedure performed. Providing information about the child’s illness is a very important aspect of contact between the parents and professional medical personnel [1,2]. Obtaining full information reinforces the woman’s sense of control and ability to make a fully informed decision independently and to control the medical stressor. Of course, patients also look for information on the Internet and in the available literature [1,3]. The decision concerning the termination of pregnancy (TOP) is complex. It depends on internal factors, such as the type of fetal defects (a lethal defect, severe fetal defect, genetic defect without structural pathologies that might make the life impossible, e.g., Trisomy 21 and the mother’s personality (family background, religious beliefs, previous mental disorders). The decision is also influenced by numerous environmental factors that may prove to be supportive or destabilizing [2,4].

Pregnancy termination is a stressful experience, which patients—depending on their adaptation skills—cope with in different ways. The process includes cognitive and behavioral actions aimed at changing the individual circumstances for the better. Coping strategies most frequently mentioned in literature with regard to TOP are: conversation (with the partner, family, friends, psychologist, physician), internalization of one’s feelings, participation in support groups, psychotherapy, seeking information (in the literature, on the Internet, on television), denial and repression, concentrating on one’s children, trying to conceive again quickly, seeking spiritual support, waiting, memorializing rituals, going on holiday with the partner, spending time with one’s children [1,5]. Patients who have undergone TOP often rebuild their sense of control by limiting social contacts and distancing themselves from others [6,7].

In their paper from 1985, Cohen & Wills distinguished between four types of social support: appraisal support, informational support, emotional support and instrumental support [8]. In the present study, appraisal support is understood as acceptance by the partner, family, community and personnel performing the procedure. In the test group, informational support was mainly provided by professional personnel, i.e., the physician, psychologist, geneticist as well as written medical information. Emotional support included: the partner, family and friends, and, to a lesser degree—a physician and a psychologist, whereas instrumental support mainly included a properly performed procedure, hospital environment and its staff. Community support means that specific people from the woman’s closest surroundings helped her deal with difficult situations [8]. Its quality and quantity depend on the personality of the person seeking support. This is most apparent in case of people with an extrovert or neurotic personality. Social support is a network of social bonds and relations having a direct or indirect impact on the person or social network whose quality and quantity depend on the quality of the interactions between the individual and their social surroundings [9]. A very important role is played by environmental resources, understood as the characteristics of the environment in which the respondent finds herself—which serve as a buffer in the situation. Generally, they can be divided into: available social support and a sense of control over the situation. A sense of control is the belief of a given person that their actions have an impact on the course of events. The woman individually determines the quality of the difficult life experience. In fact, the sense of control determines whether a stressful situation will have a mobilizing or demobilizing effect [5].

Compared to other European countries, Polish law is one of the most restrictive in terms of indications for abortion. According to the Act of 7 January 1993, pregnancy may only be terminated if it endangers the life or health of a pregnant woman, if prenatal tests or other medical indications show a high likelihood of severe and irreversible fetal defects or an incurable disease, which is a threat to its life, or if there are reasonable grounds to suspect that the pregnancy is a result of an illegal act. In case of TOP for medical reasons, the allowable TOP period is precisely defined, until “the fetus has become capable of living independently outside the mother’s body”, i.e., until the 22nd week of pregnancy [10]. In Poland, TOP is proposed to women who received the diagnosis of severe fetal defects and abnormalities after prenatal diagnostics (USG (ultrasonography), amniocentesis, chorionic villous sampling). After the patients are thoroughly informed about the prognosis, they may choose to terminate the pregnancy. Due to ideological reasons, TOP is performed in only a few centers in the country.

In this paper the authors describe the approach of Polish professional medical personnel and the manner of executing the right to refuse to perform a procedure on the basis of the conscience clause. The affected women are referred for the procedure to large cities, especially the capital. In our center, a team consisting of the head of the clinic and three consultants confirms that TOP is indeed indicated and can be legally performed. Genetic defects, such as for example Trisomy 21, are considered to be severe or irreversible fetal defects, and finally, due to a varied spectrum of manifestations the general degree of impairment will be unknown for many years [11]. Therefore, patients have the right to terminate the pregnancy in numerous situations despite the lack of structural defects in the fetus if a chromosomal aberration was identified which might affect fetal development to a large degree. Diagnosing certain structural defects, such as heart defects (e.g., hypoplastic left heart syndrome (HLHS) [12]) may also be problematic. As far as heart defects are concerned, TOP can be performed when a consulting cardiac surgeon deems the defect surgically irreparable or if surgical treatment may result in permanent and severe handicap [12,13]. In this case the final decision regarding the presence of indications for pregnancy termination is made by a case conference at the hospital where the patient presented.

The paper specifies what forms of support and medical information women expect from professional medical personnel in Poland. It describes the approach of professional medical personnel, the scope of support they can provide and the manner of executing the right to refuse to perform a procedure on the basis of the conscience clause.

## 2. Materials and Methods

The aim of the paper is to determine the patients’ needs with regard to support provided by medical personnel and the healthcare system as well as to establish what forms of support the patients expect from their partner, family and people in their surroundings to experience their period of grief in the least traumatic way.

Two detailed hypotheses were established:

1. Social support, noticed and received, plays a protective role in the process of deciding on terminating the pregnancy.
2. A sense of control is an important factor in the process of deciding on terminating the pregnancy.

At the initial stage, consent was obtained from the Bioethics Committee to carry out the study (ethical approval code: 78/PB/2014). A board composed of four members (head of the department and three specialists) verified the eligibility of patients requesting termination for medical reasons at the Department of Obstetrics and Gynecology, Centre of Postgraduate Medical Education. After the verification of the medical grounds for termination in accordance with Polish Act on Family Planning, Human Embryo Protection and Conditions of Permissibility of Abortion of 7 January 1993 [10], all the patients admitted to the hospital between June 2014 and May 2016 were asked to complete an anonymous survey consisting of sixty questions. The patients were recruited prospectively. A doctor or a midwife asked the patient to complete the survey. To eliminate the medical staff’s impact on the responses, the patients completed the survey in private (during their hospital stay). The termination procedure was performed afterwards. Surveys were returned at the time of discharge from the hospital.

The survey consisted of six sections: general information, general medical interview, pregnancy-based medical interview, religion, outlook on life, support and moral dilemmas. Some of the responses were provided on a five-point Likert scale (1—strongly agree, 5—strongly disagree). The questionnaire contained demographic data and information about the scope of medical information provided, expected forms of support and dilemmas encountered while making the decision. It took the patients around thirty minutes to complete one questionnaire. The physician provided assistance in case of any doubts. In total, one hundred and fifty surveys were collected. Statistical analysis was performed using Statistica software.

Due to the subject of the study, the majority of variables were measured on a nominal scale. Therefore, descriptive statistics and descriptions were used. In the majority of cases, while examining the strength of the patients’ beliefs, especially on the 5-point Likert scale, Spearman’s rank correlation coefficient was used to measure the strength of the correlation. All the dependencies emerged in the cross-tabulation and division tables. Therefore, there were no indications for more advanced calculations.

Pregnancy terminations at the Department of Obstetrics and Gynecology are performed using Misoprostol administered vaginally—maximally five doses depending on the term of the pregnancy and the medical interview (in case of status post C-section or surgery on the uterine muscle, the dosage is cut in half) [14]. If ineffective, the procedure is repeated the next day. If the pharmacological method proves ineffective, a Foley catheter is used to induce miscarriage by mechanically widening the cervix, or possibly oxytocin is administered through an intravenous injection.

## 3. Results

The average time of diagnosing a fetus defect was week 15.6 of pregnancy, whereas the average time of termination was week 18.0 of pregnancy. The time of the procedure is mainly due to the need to wait for traditional genetic tests where the average waiting time is at least two weeks.

In our study group, TOP was performed in only four isolated cases of heart defects. Genetic defects constituted half (50.7%) of the diagnosed problems, followed by malformation syndromes (13.3%), and defects of the central nervous system (15.3%). Out of the genetic defects, trisomies were the most common (including Trisomy 21 (42%), Trisomy 18 (23%) and Trisomy 13 (8%)), followed by triploidies (15%). Minor isolated structural pathologies without the confirmation of a genetic defect, e.g., club hands, radial agenesis, agenesis of corpus callosum, were not indications for the TOP.

### 3.1. Community Support in Pregnancy Termination

A vast majority of the respondents said that the opinion of people in their surroundings did not matter to them. Only 5% of women said that the opinion of other people in their surroundings was important to them, which is reflected in the high median and dominant (Table 1).

**Figure 1:**
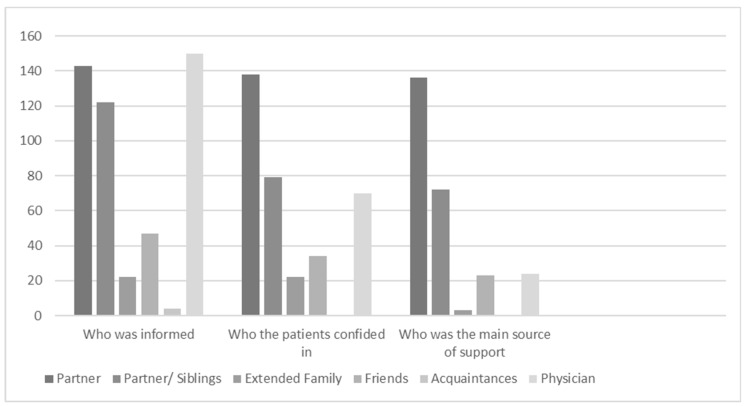
Participants in the process of deciding on pregnancy termination.

The majority of patients believe that the Polish society is intolerant when it comes to pregnancy termination, whereas less than 9% believe that the Polish nation is tolerant. However, only 23% of the respondents were afraid of being stigmatized by the society. It should be noted that the mode was close to the “no opinion” response variant. This is probably due to the fact that not many women planned to inform people in their surroundings about the termination. They only shared such information with their closest family members (Figure 1).

Even though the material standard of life of the majority of the respondents is medium, they said they would not be financially able to look after a sick child (45%). They believe that the state does not provide sufficient social support in that regard (81%).

The majority of the respondents did not indicate that they wanted to get in touch with support groups for people who had gone through similar experiences (64%). Of course, they provided the answers while undergoing the procedure, so their opinion might change afterwards, during recovery. The parents from our study population chose not to consult with parents of children with similar defects who did not terminate the pregnancy.

### 3.2. Support from the Partner and Family in Pregnancy Termination

The majority of the respondents, who met with understanding and acceptance of their decision, sought support from their closest family members (partner, parent or sibling) (Figure 1). 84% of the patients said that their partner and family supported their decision. Only less than 3% of the patients said that their family were strongly against. Only 9% of patients said that one person from their closest surroundings with whom they had spoken about the termination was against their decision. The main support group in case of termination is the family rather than professional medical personnel. However, it should be pointed out that the patients did not inform extended family or other people in their surroundings about the termination. They only shared the information with the group of people who they felt supported by. Technical support was provided by professional medical personnel, while moral support was provided by the closest family members.

Practically, all the patients had spoken to someone before making the decision to terminate the pregnancy. In most cases it was their partner (96%) and parents (55%) (Figure 1). Few patients had decided to speak to a psychologist (5%). The majority of the respondents (96%) also said that the partner’s opinion on termination was very important to them. 8% of the respondents did not take their partner’s opinion into consideration. The significance of the partner as a co-decision-maker is also suggested by median 1.0 and mode 1.0 with a slight standard deviation. The partner was also the greatest source of support for the patients (93%) (Figure 1) and supported their decision (91%). Other persons providing support to the patients included the closest family members (49%) and to a small degree a psychologist (4%) and a physician (12%) (Figure 1). Only a few patients said they had no support (2%).

### 3.3. Support Provided by Professional Medical Personnel and Informational Support

**Figure 2:**
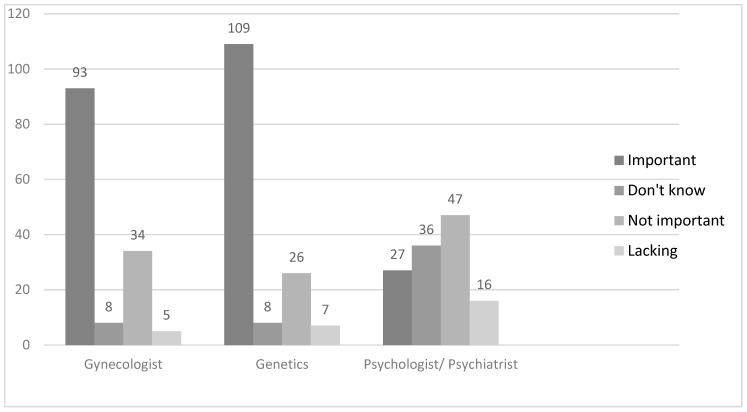
Importance of contact with professional medical staff in the process of deciding on pregnancy termination.

Nearly one-third (31%) of the respondents had not spoken to the attending gynecologist about their decision to terminate the pregnancy, and 48% of the respondents did not ask if their attending physician performed termination procedures. The majority of the respondents indicated that the gynecologist’s opinion was very important to them (Figure 2) (median 2.0, mode 1.0, standard deviation 1.45), but they did not necessarily mean the attending gynecologist.

Perhaps a lack of trust towards the attending gynecologist arises from the fear of social ostracism and the attitude of the physician aimed at avoiding the problem. The patients also stated that the geneticist’s opinion was very important in the decision-making process (Figure 2), (median 1.0, mode 1.0, standard deviation 1.37). However, it should be noted that psychologists or psychiatrists played a minor role in the decision-making process (Figure 2).

### 3.4. Medical Concerns

Patients indicated that medical information was very important in the decision-making process (Table 2).

Only 10% of the patients reported having no medical information. The lack of medical concerns suggests that the patients had sufficient information, which made them feel comfortable and gave them a sense of control in the decision-making process. The importance of the information provided was also noticed when it comes to returned surveys. The first 60 surveys were given out by the physician who is the main author of the project, who explained the nature of the study in detail, achieving the return rate of 100%. Subsequent surveys were given to the patients by a random physician or a midwife at the time of admission to the hospital. In that case, the return rate reached 42.1%. In total, the return rate was 62.5%. This also shows the importance of a personal approach to patients.

Female patients participating in the study appreciated the support and empathy received from the medical personnel performing the procedure. However, only 5% of patients said that they had confided in a psychologist, and 44% in a physician. The patients who had talked to the attending physician about the termination were also more willing to talk to their partner and parents. The patients who did not talk to the attending physician spoke to their friends, extended family, a psychologist or a physician more rarely. The result may suggest a more introvert personality. Moreover, fewer of those patients sought support from extended family, friends or a physician.

## 4. Discussion

Numerous external factors influence the decision-making process when it comes to pregnancy termination: legislation, healthcare system, scope of medical insurance, access to healthcare services, activity of support groups, social status and access to medical knowledge [15,16,17]. The decision to terminate or keep a pregnancy depends on various factors, the chief ones being the psychological constitution of the mother, ideology, personal beliefs, concerns and doubts about the diagnosis, as well as hope that the child will survive [17,18].

While making a decision, on the one hand, the woman feels in control as she can make the decision whether to give birth to a sick child and, on the other hand—she has to make a decision she never wanted to make [19]. Irrespective of how difficult that decision may be, the majority of women—as shown by this study as well as by studies carried out by other researchers—say they made the right decision [20].

Couples analyze the social consequences of having a sick child. Many women also consider financial limitations associated with rehabilitation and hospital treatments [21,22], which was also reported by the patients of the Department of Obstetrics and Gynecology, Center for Postgraduate Medical Education. The scale of the problem was marked by very strong objections raised by parents of disabled children who feel neglected and abandoned by state authorities. Such parents have to give up important areas of their lives to provide the child with proper care [23,24].

One important aspect of the decision-making process is the view on the support provided by the partner [25,26]. More than half of the patients (55%) who had decided to terminate pregnancy due to Trisomy 21 diagnosis said that the reason was that they were afraid of the impact of giving birth to a sick child on their relationship with the partner [26]. In addition, 38% of the patients reported that their fears were related to a disagreement with their partner about the termination. In this study, a high level of acceptance from the partner with regard to pregnancy termination was observed (nearly 100%). The patients did not report their partner being against their decision; in fact, the partner was the main source of support. Another important aspect was a strong position of the partner as the decision-maker and authority for the female patients in our study. Such significant correlations may only be found in scientific reports from India, Nepal, and Bangladesh [27,28,29].

A study by Antenatal Results and Choices—an organization providing assistance before, during and after prenatal tests in the United Kingdom—indicated that the most important aspect of advisory services is to provide full exhaustive information about the prognosis for the fetus and the technique of performing the procedure, which helps the parents believe that they did everything they could in such a difficult situation [30]. The benefits of deciding, and the resulting sense of control, is emphasized in an American study on the satisfaction with the possibility to choose the method of termination (surgical or pharmacological termination). Patients’ sense of control contributed not only to higher satisfaction with the decision but also to long-term coping and grief resolution [31].

Another important issue is the process of informing the patient about the possibility of terminating the pregnancy and the place where such a procedure may be performed [32]. In Poland, the access to genetic advisory services and facilities where termination procedures are performed depends to a large extent on the place of residence [33]. Some patients do not undergo ultrasound scans and are not aware of being able to terminate a pregnancy in case of a sick fetus [34]. With regard to providing information about the prognosis and available options, some doctors invoke the conscience clause and do not inform patients about the possibility of terminating the pregnancy. Many women undergo terminations outside Poland, especially if there are doubts whether there are sufficient grounds for termination. The high rate of survey returns (62.5%) is related to the patients’ gratitude for being able to have the procedure performed. Twenty-one percent of the patients used the final notes section to express their gratitude and share positive opinions on the personnel working at the hospital.

In the study described in this paper, the patients did not report any medical concerns or any issues with medical personnel interfering with independent decision-making process. However, it should be noted that they only asked their closest family members or physicians directly involved in diagnosing the defect and performing the termination procedure for their opinion.

The level of stress experienced during a termination procedure depends on the patient’s basic resources (personality, values, support from partner and family) [21]. Negative factors include: previous psychiatric issues, planned and wanted pregnancy, pressure from people in the surroundings, no social support, a personality with a higher tendency to react negatively to stress (low self-esteem, pessimism, low sense of control). The same factors may cause mental disorders in women who decide to continue with the pregnancy [35,36]. Literature mentions protective factors, such as: support from the partner and close family, no past mental illnesses, higher education, no medical concerns and young age [37]. Regardless of the evidence described above, a higher risk of emotional complications among the patients participating in the study may probably be found in case of introvert patients, who had not sought support from the attending physician or people in their surroundings. According to the latest study by Kerns et al. (2018) conducted among online support groups higher decision satisfaction and shared decision making is associated with lower feeling of sadness and less frequent occurrence of post-traumatic stress disorder [31].

In a study involving 997 married couples from Nepal who decided to undergo an abortion, the main factor in the decision-making process was the husband and professional medical personnel [28]. A similar role in the decision-making process was played by husbands in a study conducted in India. In case of young women living with their husband and mother-in-law, the household members made the decision on abortion [29]. In some countries, the husband is the ultimate decision-maker, but the support group for women deciding on abortion includes neighbors, sisters-in-law, friends, and professional medical personnel [38]. The study discussed in this paper also indicated a significant role of the partner in the decision-making process. In a study by Major, the patients expressed a will to get in touch with professional medical personnel, support groups and organizations supporting women who had undergone a termination. They believed it may help them recover faster. The study indicates that social support is a key element of the recovery process. However, the patients stressed that talking about termination was a very difficult experience for them [39]. The authors suggested that the rationalization of the decision may make it easier for women to overcome negative feelings and sadness, especially social isolation. They stressed the importance of support groups for women after abortion. The majority of couples (72%) reported the need for contact with other people who had experienced a similar problem [39]. In a study from China, a group of women and their families, who were included in the intervention group and received psychological assistance during and after TOP, proved to have the lowest rate of subsequent psychological complications, such as post-traumatic stress and depression [40]. In the Polish reality, due to high social stigmatization, women tend to avoid support groups and contact with a psychologist, as they would then have to admit to having undergone termination. Such an attitude may strengthen the feeling of sadness, loneliness and blaming themselves for the termination. A study by Speckhard and Major et al. showed that women who display an avoidance attitude as a strategy of dealing with stress encounter growing emotional problems over time even if they initially seemed perfectly adjusted to the situation [39,41]. An important role in the emotional recovery process is played by support groups and therapeutic groups. They allow them to verbalize emotions and reduce the sense of isolation as well as to exchange views. The patients did not have a significant need to get in touch with someone with similar experiences or to talk to a psychologist. The main support group for them was the partner and closest family members. This model is usually found in societies with a multigenerational family model. A study on the psychological consequences experienced by midwives participating in termination procedures shows that a higher index of perceived emotional and instrumental support correlates with a lesser likelihood of developing post-traumatic stress disorder [42]. A survey was conducted at a private clinic in Houston to determine the need for support among 51 patients who had decided to terminate a pregnancy due to fetal defects. It was carried out during the procedure, six weeks and three months after the procedure and it showed that women experienced the need for support in different ways. Many women stated that they were not prepared mentally for the consequences of the procedure and they needed long-term professional assistance [43]. The main support group and co-decision-makers were the partner (96%) and family (88%), as well as the geneticist and friends. Only 5.9% of the patients said that they were going to seek help from a psychiatrist, psychologist or a trusted person. 15.7% reported no need for support. The survey was conducted at a private facility, where the patients were mainly highly-educated women, which excludes it from being representative of the whole country. However, the test group was similar in terms of their educational background to the group described in this paper. The authors recommended women undergoing termination to participate in a psychological consultation in order to prepare themselves for subsequent emotional consequences of the procedure [43]. Our patients represented a similar community group and also did not want to continue the cooperation, which probably resulted from social stigmatization. Research showed that women do not reveal the information of pregnancy termination for fear of social stigmatization [2,20]. Studies conducted in Germany demonstrated that patients living in the eastern region, which is more liberal than the western one, reported a lower sense of being stigmatized [44,45].

While terminating pregnancy women are frequently unaware that they will later require psychological support due to a delayed sense of sadness that they experience. Usually, the first symptoms appear within four months up to a year from the procedure. A survey conducted among 2945 women whose child had Trisomy 21 showed that their main source of emotional support came from groups for mothers of children with the same disease [46]. Parents are aware that they will remember the decision they made for the rest of their life. Usually, they seek support and contact with others two or three months after the procedure. This correlates in time with the anniversary of the child’s death and with the family’s and friends’ wish to go back to normal [30].

It should be noted that the majority of such studies are conducted among members of support groups for women who have undergone termination, which may have a significant influence on the results. Support groups for people who experienced an abortion in Poland are mainly run by religious organizations. Asplin et al. and Salvesen et al. indicated that women need systemic support later on at different stages of their life [47,48]. Mailing groups and online groups seem to be a good forward-looking solution, as they offer anonymity, ease of access and a sense of community. According to the authors, the dangers related to virtual forms of support include creating an unhealthy obsession and sharing incorrect medical information. The perfect solution would be to have a moderator with a degree in psychology, who could identify incorrect adaptation mechanisms in the process of coping with the problem. In the literature, we can find comments about a lack of professional support after a procedure [47,48].

Women commonly feel lonely and abandoned with this difficult problem [49]. However, it should be pointed out that the feeling of loneliness may occur in patients who isolated themselves from their surroundings. In the study described herewith, the patients did not feel the need to become members of support groups or to get in touch with people with similar experiences. The patients did not want to hide from the world, which is evidenced by the fact that the majority of them did not ask to take half of their maternity leave, which they have a statutory right to, i.e., several months of paid leave, and instead only asked for two to four weeks of medical leave. Afterwards, they were planning to return to work. This may result from the fact that they probably did not plan to admit to a miscarriage at work. Abortion creates the atmosphere of shame and secrecy. Many women are afraid of being judged. A study from Israel showed termination as a taboo hidden behind a wall of silence [50]. The majority of the patients only shared a section of the story with people in their surroundings—for example, saying that they miscarried. The Polish society stigmatizes both women who terminated a pregnancy and families with disabled children. The stigmatization creates negative cognitive, emotional and behavioral patterns, which may affect the social, psychological and biological functioning of the mother and her family. Women who internalize the feeling of stigmatization (blame themselves or think that they must have a moral deficit) bear a higher risk of psychological complications developing at a later time.

Patients appreciate a nonjudgmental attitude of professional medical personnel [47,51]. The participants of the study described herein gave very positive feedback to the holistic approach of professional medical personnel and to the amount of medical information received. The right approach to the patient significantly helps eliminate stress and trauma associated with the procedure. In the literature, we can find accounts presented by many women talking about a lack of professional support after the procedure, which may have been a result of personal beliefs of medical personnel [47].

The present study demonstrates the problem of the TOP in Polish cultural, religious, ethical, and political reality. Several years before, the TOP problem concerning cultural aspects in Germany was presented in a study by Erikson [45]. A vast majority of women (90–100%) undergoing prenatal diagnostics decided to terminate the pregnancy after receiving the prenatal diagnosis of fetal defects, even if the defects were not severe. Erikson’s observations showed that, similarly to Polish patients, women in Germany separated religious issues from decisions concerning procreation. The author presented a concept invoked by pro-life groups that viewed the TOP as neo-eugenic approach comparable with Holocaust. Therefore, a question arises whether the TOP due to fetal defects may be considered as eugenic abortion, especially with such broad indications for the TOP. The stigmatization of disabled children is another issue that is commonly neglected or considered to be uncomfortable to discuss [52]. According to Erikson, women mentioned situations in which they were asked by strangers whether the defect in their child could not have been diagnosed prenatally [45].

### Study Limitations

A great number of scientific papers on abortion and termination bear significant methodological limitations due to the sensitive character of the subject at hand.

The first issue is the lack of a control group. It is difficult to select women with similar psychological traits and a similar social situation, from a similar cultural environment. There are few patients who decide to continue with the pregnancy that could be the control group. Women from countries with restrictive laws and religion are less willing to talk about abortion, which leads to a low response rate some time after the procedure. Women from places where abortion is stigmatized very often do not want to go back to talking about that difficult subject. Therefore, no further attempt was made to interview them again, once more time has passed since the procedure. It is difficult to choose the appropriate time for subsequent interviews, as there are no clear indications of when such an interview should be carried out.

The study was conducted at one facility situated in the capital of the country. As termination is not commonly available, the test group may not be representative for the rest of Poland. There are few centers which perform termination procedures in Poland, which is why the study was limited to only one facility performing nearly half of all the procedures in the Mazowieckie Province and one-sixth of all the procedures in the country. A strong point of the study is a high number of participants (150) with regard to the number of procedures performed in the country and a high survey return rate with regard to the sociocultural situation in Poland (62.5%).

## 5. Conclusions

Decision concerning the TOP is complex and depends on numerous internal and external factors. Women who decided to terminate the pregnancy positively assessed informational support which was provided by professional personnel in Poland (the physician who diagnosed the defect, psychologist, geneticist). Notably, a lot of women do not consult their decision with the attending gynecologist, which probably arises from the fear of stigmatization. The above results from the fact that numerous physicians in Poland invoke the conscience clause and refuse to terminate pregnancies or do not inform the patient about the possibility of the TOP. Women do not take the society’s opinion into consideration. Such an approach and the resultant internalization of stigma may generate mental disorders in the future which was reported in the professional literature. Additionally, unwillingness to contact support groups or a psychologist is not caused by the fact that we differ from other nations, but rather from the fear of being stigmatized by the society. Strong emotional support from the partner and the closest family, who agreed with the women’s decision in almost 100% of cases, is an optimistic phenomenon.

The present authors believe that the study will indicate the weakest links, i.e., no possibility to express one’s trauma and strong fear of social stigmatization, in the process of mental recovery following TOP procedures in Poland.

Authors are planning a further study about how to improve TOP information support from medical professionals that deal with women who are deciding to terminate their pregnancy and a study in a group of women who have decided to continue their pregnancy despite the possibility of termination due to the fetal abnormalities - their psychological aspects and opinions about medical professionals support.

This study is the first attempt to tackle this difficult topic, which is not discussed openly and without reservations by the Polish population. It reveals a major social problem which we need to be aware of and attempts should be undertaken to solve it.

## Figures and Tables

**Table 1:** Importance of the opinion of people in close surroundings on pregnancy termination, statistical view.

Is the Opinion of People in Close Surroundings about TOP Important to You?	*n*	%
Very important	5	3.33
Important	3	2
It depends	29	19.33
Less important	18	12
Not important	87	58
Incomplete forms	8	5.33

TOP: termination of pregnancy.

**Table 2:** The significance of medical information in the decision-making process, statistical perspective.

How Important are Medical Information about the Child’s Defect and TOP?	*n*	%
Very important	87	58
Important	25	16.67
It depends	8	5.33
Less important	1	0.67
Not important	20	13.33
Incomplete forms	9	6

## References

[1] Makara-Studzińska M. (2012). Komunikacja z Pacjentem.

[2] Cockrill K., Upadhyay U.D., Turan J., Greene Foster D. (2013). The stigma of having an abortion: Development of a scale and characteristics of women experiencing abortion stigma. Perspect. Sex. Reprod. Health.

[3] Alkaabi M.S., Alsenaidi L.K., Mirghani H. (2015). Women’s knowledge and attitude towards pregnancy in a high-income developing country. J. Perinat. Med..

[4] Frederico M., Michielsen K., Arnaldo C., Decat P. (2018). Factors influencing abortion decision-making processes among young women. Int. J. Env. Res. Public Health.

[5] Desrochers J.N. (2011). The psychological impact of termination of pregnancy for fetal anomaly of the male partner. Master’s Thesis.

[6] Bryar S.H. (1997). One day you’re pregnant and one day you’re not: Pregnancy interruption for fetal anomalies. J. Obstet. Gynecol. Neonatal. Nurs..

[7] Rillstone P., Hutchinson S.A. (2001). Managing the reemergence of anguish: Pregnancy after a loss due to anomalies. J. Obstet. Gynecol. Neonatal. Nurs..

[8] Cohen S., Wills T.A. (1985). Stress, social support, and the buffering hypothesis. Psychol. Bull..

[9] Altshuler A.L., Whaley N.S. (2018). The patient perspective: Perceptions of the quality of the abortion experience. Curr. Opin. Obstet. Gynecol..

[10] Polish Government (1993). Act on Family Planning, Protection of the Human Fetus and Conditions for the Admissibility of Termination of Pregnancy.

[11] Marchal J.P., Maurice-Stam H., Houtzager B.A., Rutgers van Rozenburg-Marres S.L., Oostrom K.J., Grootenhuis M.A., van Trotsenburg A.S.P. (2016). Growing up with down syndrome: Development from 6 months to 10.7 years. Res. Dev. Disabil..

[12] Walsh M.J., Verghese G.R., Ferguson M.E., Fino N.F., Goldberg D.J., Owens S.T., Pinto N., Zyblewski S.C., Quartermain M.D. (2017). Counseling practices for fetal hypoplastic left heart syndrome. Pediatr. Cardiol..

[13] Ntiloudi D., Zegkos T., Koutsakis A., Giannakoulas G., Karvounis H. (2017). Pregnancy in patients with congenital heart disease: A contemporary challenge. Cardiol. Rev..

[14] Morris J.L., Winikoff B., Dabash R., Weeks A., Faundes A., Gemzell-Danielsson K., Kapp N., Castleman L., Kim C., Ho P.C. (2017). Figo’s updated recommendations for misoprostol used alone in gynecology and obstetrics. Int. J. Gynaecol. Obstet..

[15] Abortion Statistics England and Wales 2014: Summary Information From Abortion Forms Returned to the Chief Medical Officers. https://assets.publishing.service.gov.uk/government/uploads/system/uploads/attachment_data/file/433437/2014_Commentary__5_.pdf.

[16] De Crespigny L.J., Savulescu J. (2008). Pregnant women with fetal abnormalities: The forgotten people in the abortion debate. Med. J. Aust..

[17] Abi Tayeh G., Jouannic J.M., Mansour F., Kesrouani A., Attieh E. (2018). Complexity of consenting for medical termination of pregnancy: Prospective and longitudinal study in paris. BMC Med. Ethics.

[18] Herold S., Kimport K., Cockrill K. (2015). Women’s private conversations about abortion: A qualitative study. Women Health.

[19] Rocca C.H., Kimport K., Roberts S.C., Gould H., Neuhaus J., Foster D.G. (2015). Decision rightness and emotional responses to abortion in the united states: A longitudinal study. PLoS ONE.

[20] Lafarge C., Mitchell K., Fox P. (2014). Termination of pregnancy for fetal abnormality: A meta-ethnography of women’s experiences. Reprod. Health Matters.

[21] Steinberg J.R., Russo N.F. (2008). Abortion and anxiety: What’s the relationship?. Soc. Sci. Med..

[22] Bijma H.H., van der Heide A., Wildschut H.I. (2008). Decision-making after ultrasound diagnosis of fetal abnormality. Reprod. Health Matters.

[23] Associated Press Parents of Disabled Polish Children Keep up Protests. https://www.washingtonpost.com/world/europe/parents-of-disabled-polish-children-keep-up-protests/2018/04/27/dc48ec18-4a0b-11e8-8082-105a446d19b8_story.html?utm_term=.5ea6b2fb1995.

[24] Associated Press Poland: Parents, disabled children end parliament protest. https://www.usnews.com/news/world/articles/2018-05-27/poland-parents-disabled-children-end-parliament-protest.

[25] Korenromp M.J., Page-Christiaens G.C., van den Bout J., Mulder E.J., Visser G.H. (2006). Is there pressure from society to terminate pregnancy in case of a fetal anomaly?. Prenat. Diagn..

[26] Korenromp M.J., Page-Christiaens G.C., van den Bout J., Mulder E.J., Visser G.H. (2007). Maternal decision to terminate pregnancy in case of down syndrome. Am. J. Obstet. Gynecol..

[27] Ahmed S., Parveen S.D., Islam A., Khanum P.A. (1997). Induced Abortion: Results From Two Rural Areas of Bangladesh.

[28] Puri M., Ingham R., Matthews Z. (2007). Factors affecting abortion decisions among young couples in Nepal. J. Adolesc. Health.

[29] Ganatra B., Hirve S. (2002). Induced abortions among adolescent women in rural Maharashtra, India. Reprod. Health Matters.

[30] Fisher J. (2008). Termination of pregnancy for fetal abnormality: The perspective of a parent support organisation. Reprod. Health Matters.

[31] Kerns J.L., Light A., Dalton V., McNamara B., Steinauer J., Kuppermann M. (2018). Decision satisfaction among women choosing a method of pregnancy termination in the setting of fetal anomalies and other pregnancy complications: A qualitative study. Patient. Educ. Couns..

[32] Gould H., Perrucci A., Barar R., Sinkford D., Foster D.G. (2012). Patient education and emotional support practices in abortion care facilities in the united states. Women. Health Issues.

[33] Eriksson M. (2000). Reproductive Freedom in the Context of International Human Reproductive Rights and Humanitarian Law.

[34] Liamputtong P., Halliday J.L., Warren R., Watson F., Bell R.J. (2003). Why do women decline prenatal screening and diagnosis? Australian women’s perspective. Women Health.

[35] Speckhard A.C., Rue V.M. (1992). Postabortion syndrome: An emerging public health concern. J. Soc. Issues.

[36] Speckhard A., Mufel N. (2009). Uniwersalne Reakcje na Aborcję? Więź, Trauma i żal u Kobiet z Doświadczeniem Aborcji, (w:) Aborcja. Przyczyny, następstwa, terapia.

[37] Lafarge C., Mitchell K., Fox P. (2013). Women’s experiences of coping with pregnancy termination for fetal abnormality. Qual. Health Res..

[38] Puri M., Chaudhary P. Situational Analysis Of Unsafe Abortion in Nepal. https://www.figo.org/sites/default/files/NEPAL.doc.

[39] Major B., Richards C., Cooper M.L., Cozzarelli C., Zubek J. (1998). Personal resilience, cognitive appraisals, and coping: An integrative model of adjustment to abortion. J. Pers. Soc. Psychol..

[40] Sun S., Li J., Ma Y., Bu H., Luo Q., Yu X. (2018). Effects of a family-support programme for pregnant women with foetal abnormalities requiring pregnancy termination: A randomized controlled trial in china. Int. J. Nurs. Pr..

[41] Speckhard A. (1985). Psycho-Social Stress Following Abortion.

[42] Banasiewicz J. (2013). Konsekwencje Psychologiczne Występujące u Położnych Uczestniczących w Zabiegach Przerwania Ciąży. Ph.D. Thesis.

[43] Ramdaney A., Hashmi S.S., Monga M., Carter R., Czerwinski J. (2015). Support desired by women following termination of pregnancy for a fetal anomaly. J. Genet. Couns..

[44] Hanschmidt F., Treml J., Klingner J., Stepan H., Kersting A. (2018). Stigma in the context of pregnancy termination after diagnosis of fetal anomaly: Associations with grief, trauma, and depression. Arch. Womens Ment. Health.

[45] Erikson S.L. (2003). Post-diagnostic abortion in germany: Reproduction gone awry, again?. Soc. Sci. Med..

[46] Skotko B.G. (2005). Prenatally diagnosed down syndrome: Mothers who continued their pregnancies evaluate their health care providers. Am. J. Obstet. Gynecol..

[47] Asplin N., Wessel H., Marions L., Georgsson Ohman S. (2012). Pregnant women’s experiences, needs, and preferences regarding information about malformations detected by ultrasound scan. Sex. Reprod. Heal..

[48] Salvesen K.A., Oyen L., Schmidt N., Malt U.F., Eik-Nes S.H. (1997). Comparison of long-term psychological responses of women after pregnancy termination due to fetal anomalies and after perinatal loss. Ultrasound Obstet. Gynecol..

[49] Jayaweera R.T., Ngui F.M., Hall K.S., Gerdts C. (2018). Women’s experiences with unplanned pregnancy and abortion in kenya: A qualitative study. PLoS ONE.

[50] Leichtentritt R.D. (2011). Silenced voices: Israeli mothers’ experience of feticide. Soc. Sci. Med..

[51] Kerns J., Vanjani R., Freedman L., Meckstroth K., Drey E.A., Steinauer J. (2012). Women’s decision making regarding choice of second trimester termination method for pregnancy complications. Int. J. Gynaecol. Obstet..

[52] Rossler W. (2016). The stigma of mental disorders: A millennia-long history of social exclusion and prejudices. EMBO Rep..

